# Supplementary material for: Ionomic Combined with Transcriptomic and Metabolomic Analyses to Explore the Mechanism Underlying the Effect of Melatonin in Relieving Nutrient Stress in Apple
Source: Int J Mol Sci. 2022 Aug 30;23(17):9855. doi: 10.3390/ijms23179855 (PMC9456084; doi:10.3390/ijms23179855)
Supplement: Supplementary file 1 [file ijms-23-09855-s001.zip › ijms-1814653-supplementary.pdf]

Table S1 Effects of melatonin treatment on concentration of macro- and microelements in roots, stems, and leaves of *Malus hupehensi*s. Data are means ± SD of 4 replicate samples. Values not followed by same letter denote significant differences by Tukey's multiple range tests (P<0.05)

Element	Tissue	CK	MCK	ST	MST
N(mg/g)	Root	17.22±0.83b	19.32±1.77a	14.71±0.70c	16.61±0.41b
	Stem	11.45±0.60a	11.57±0.18a	10.57±0.31b	11.82±0.17a
	Leaf	23.28±0.95b	26.53±1.60a	19.86±0.76c	22.95±0.89b
P(mg/g)	Root	15.11±0.84ab	15.40±0.66a	12.99±0.19c	14.03±0.71bc
	Stem	7.47±0.23b	8.42±0.39a	6.32±0.36c	7.61±0.30b
	Leaf	15.01±0.11b	16.00±0.19a	12.94±0.04d	13.45±0.46c
K(mg/g)	Root	14.98±0.08a	14.73±0.31a	12.15±0.03c	13.25±0.46b
	Stem	7.84±0.06a	7.74±0.18a	6.39±0.09c	6.95±0.05b
	Leaf	13.74±0.13a	13.82±0.12a	11.70±0.10b	13.47±0.37a
Ca(mg/g)	Root	11.38±0.54b	13.35±0.11a	7.04±0.20c	7.14±0.36c
	Stem	5.70±0.05b	7.00±0.03a	5.35±0.08c	5.44±0.11c
	Leaf	7.83±0.03c	8.17±0.02b	8.50±0.09a	8.52±0.11a
Mg(mg/g)	Root	1.46±0.04b	1.57±0.01a	1.39±0.03c	1.61±0.01a
	Stem	0.78±0.01b	0.75±0.01c	0.98±0.02a	0.79±0.01b
	Leaf	1.52±0.1b	1.69±0.03a	1.49±0.01b	1.65±0.02a
Fe(μg/g)	Root	1083.92±70.02b	1261.98±63.19a	986.36±28.31d	1154.61±31.82c
	Stem	632.31±61.71 b	1100.97±42.99a	638.91±21.00 d	724.23±25.94c
	Leaf	1366.90±76.10 b	2045.00±22.14a	866.45±74.96 d	1055.85±122.84c
Mn (μg/g)	Root	556.40±45.5b	846.16±35.96b	598.53±37.79c	574.22±13.33b
	Stem	243.01±12.71c	362.27±3.69a	270.36±4.15b	282.93±2.56b
	Leaf	759.20±32.31a	619.38±3.07b	282.62±17.05d	627.46±6.87b
Cu(μg/g)	Root	157.96±0.83a	157.25±6.52a	104.98±4.30c	114.89±3.37b
	Stem	59.10±0.97a	56.11±0.42a	46.26±3.55c	49.89±0.88b
	Leaf	159.01±2.95	142.74±2.70	87.06±4.69	81.60±1.78
Zn(μg/g)	Root	147.72±4.61c	316.11±10.48a	124.02±1.30d	159.38±4.01b
	Stem	60.21±0.81a	60.07±0.27a	23.74±0.32c	46.61±1.92b
	Leaf	107.05±1.00a	87.47±1.07c	111.07±4.12a	98.28±1.47b
B(μg/g)	Root	28.57±1.31b	43.04±0.45a	27.61±2.40b	25.97±0.86b
	Stem	37.45±1.25a	35.38±2.45b	27.61±2.41a	35.06±2.84a
	Leaf	51.36±0.84a	48.20±1.56b	42.68±1.43c	46.24±1.40b

Table S2 Statistical table of sequencing output

Sample	Raw Reads	Clean Reads	Reads mapped	Q20 (%)	Q30 (%)	GC Content (%)
CK1	46467410	45260868	39336358(86.91%)	98.14	94.52	46.48
CK2	49431888	48178138	41128176(85.37%)	98.13	94.56	46.4
CK3	48230204	46885336	40067098(85.46%)	98.23	94.7	46.22
MST1	51626096	49609060	42484516(85.64%)	98	94.32	47.03
MST2	50840034	49127864	41040011(83.54%)	97.91	94.09	46.8
MST3	49428100	47355980	40065422(84.60%)	98.03	94.38	46.78
ST1	53421566	52239700	44490152(85.17%)	98.25	94.76	46.4
ST2	46875062	45494316	39180879(86.12%)	98.1	94.4	46.49
ST3	56789648	55087670	46531427(84.47%)	98.05	94.41	46.54

Table S3 Statistical table of differential genes

group	total	down	up
MST/CK	285	145	140
ST/CK	111	64	47
MST/ST	138	70	68

Table S4 Candidate genes related to chlorophyll, response to stress, metal ion and phosphate in apple.

Annoation	ST/CK			MST/ST		
	ID	log_2FC	p-value	ID	log_2FC	p-value
chlorophyll	MD00G1131800	1.116	0.000154919	MD05G1321000	-1.067	8.29889234004689e-10
	MD01G1187500	-2.207	1.14654360656962e-24	MD06G1134100	1.45	7.52056415863752e-11
	MD04G1164500	-4.173	2.04034E-05			
	MD04G1164600	-1.903	4.84866397403326e-21			
	MD08G1207100	-1.04	1.302375600132e-12			
	MD13G1003800	-1.021	4.51706E-06			
response to stress	MD01G1208700	1.378	2.57076974605695e-10	MD04G1117800	1.753	4.83612E-05
	MD02G1045900	-1	5.81734382444172e-13	MD05G1073200	-1.287	2.9976482686681e-14
	MD02G1076300	-1.044	2.62975965144311e-9	MD06G1085400	-1.486	2.16565E-05
	MD11G1189000	-1.186	4.93741647596455e-9	MD06G1161300	-1.931	3.85987701134797e-10
	MD11G1210800	1.874	0.000364456	MD07G1240700	1.39	5.3515E-05
	MD13G1147700	-1.314	6.03123624312019e-7	MD11G1315600	-1.513	3.45936E-05
	MD13G1172200	-1.212	2.65416E-05	MD12G1125600	1.48	0.000100574
metal ion	MD11G1069200	1.538	1.38144E-05	MD00G1187000	1.185	7.42153914835457e-9
	MD11G1246800	-1.208	6.39204080937249e-14	MD02G1185900	1.353	0.002864201
	MD12G1178200	-4.709	6.71845641223189e-7	MD04G1124400	1.12	0.000228755
	MD12G1178500	-5.196	1.51113E-06	MD05G1118500	1.259	3.89014E-05
	MD14G1086500	4.43	3.01636E-05	MD05G1144800	1.002	0.001421779
	MD14G1191800	1.459	4.33921008794752e-8	MD05G1335100	-2.156	0.00030365

	MD14G1198000	1.115	1.17375059328541e-7	MD08G1196500	-1.106	5.39106478013394e-8
	MD15G1025600	1.844	1.44032E-05	MD09G1033600	1.035	0.000829655
	MD16G1016600	-1.96	3.30203265525986e-7	MD10G1227800	1.801	0.000197607
				MD02G1145900	-1.103	5.20749359325183e-7
				MD12G1003300	-1.455	0.001022669
**phosphate**				MD07G1253900	1.332	0.003231627

Table S5 Statistical table of differential metabolites

group	total	down	up
MST/CK	14	5	9
ST/CK	11	9	2
MST/ST	16	7	9

Table S6 Statistical table of different metabolite substances in ST/CK

Compounds	Log_2FC	Type
Oxalic acid	-1.00E+01	down
3-Ureidopropionic Acid	-2.21E+00	down
L-Asparagine	-2.29E+00	down
L-Aspartic Acid	-1.24E+00	down
Muconic acid	-1.63E+00	down
2,2-Dimethylsuccinic acid	-1.23E+00	down
N-Acetylisatin	1.07E+00	up
2'-Deoxyinosine-5'-monophosphate	-1.06E+00	down
Isorhamnetin-3-O-gallate	-1.08E+00	down
Dihydromyricetin-3-O-glucoside	1.03E+00	up
Solatriose	-1.32E+00	down

Table S7 Statistical table of different metabolite substances in MST/ST

Compounds	Log_2FC	Type
Oxalic acid	1.12E+01	up
2-Aminoethanesulfonic acid	-1.43E+00	down
L-Ascorbic acid (Vitamin C)	1.23E+00	up
Dihydroresveratrol	-1.07E+00	down
Melatonin (N-Acetyl-5-methoxytryptamine)	2.02E+00	up
p-Coumaroylputrescine	-1.56E+00	down
9-Hydroperoxy-10E,12,15Z-octadecatrienoic acid	-1.41E+00	down

13S-Hydroperoxy-6Z,9Z,11E-octadecatrienoic acid	-1.81E+00	down
2-Acetyl-3-hydroxyphenyl-1-O-glucoside	1.25E+00	up
3-O-Methylquercetin	-1.29E+00	down
Cocamidopropyl betaine	1.15E+00	up
Lirioresinol A	1.18E+00	up
Syringaresinol	1.20E+00	up
Cyanidin-3-O-galactoside	1.07E+00	up
Dihydromyricetin-3-O-glucoside	-1.10E+00	down
Solatriose	1.07E+00	up

Table S8 Statistical table of different metabolite substances

**Compounds**	**ST/CK**	**MST/ST**
Oxalic acid	down	up
2-Aminoethanesulfonic acid	FALSE	down
3-Ureidopropionic Acid	down	FALSE
L-Asparagine	down	FALSE
L-Aspartic Acid	down	FALSE
Muconic acid	down	FALSE
2,2-Dimethylsuccinic acid	down	FALSE
L-Ascorbic acid	FALSE	up
N-Acetylisatin	up	FALSE
Dihydroresveratrol	FALSE	down
Melatonin	FALSE	up
p-Coumaroylputrescine	FALSE	down
9-Hydroperoxy-10E,12,15Z-octadecatrienoic acid	FALSE	down
13S-Hydroperoxy-6Z,9Z,11E-octadecatrienoic acid	FALSE	down
2-Acetyl-3-hydroxyphenyl-1-O-glucoside	FALSE	up
3-O-Methylquercetin	FALSE	down
2'-Deoxyinosine-5'-monophosphate	down	FALSE
Cocamidopropyl betaine	FALSE	up
Lirioresinol A	FALSE	up
Syringaresinol	FALSE	up
Cyanidin-3-O-galactoside	FALSE	up
Isorhamnetin-3-O-gallate	down	FALSE
Dihydromyricetin-3-O-glucoside	up	down
Solatriose	down	up

Table S9 Sequences of primers used in qRT-PCR

**Gene**	**Primer sequence (5'-3′)**
MD05G1178500 F	ATCGTCTTTAGGCGCGGTAG
MD05G1178500 R	CAGCCCATCTCAGAAGCTCC

MD10G1227800 F	CCGCCGAATGTGAATGCAAT
MD10G1227800 R	GAATCCTCTGGTGCAATCGC
MD04G1117800 F	TCGGGATACGCGAAGGAATG
MD04G1117800 R	GACCGTCAGATCGTGGATCC
MD01G1089600 F	GCTATGCCATGCAGCTTGTG
MD01G1089600 R	TCAATGCCATCAAGGGACCC
MD12G1125600 F	GTACCCGACTGTGAGTGAGG
MD12G1125600 R	GGAGGACTATCGGAGCACAG
MD03G1251400 F	AACCCTAGCCCAAACATCCG
MD03G1251400 R	ACTGAACCATCCAAGGGCCAG
MD05G1132400 F	TGGGAAGTACGAAGGTCCTCA
MD05G1132400 R	GCTCCAATTACCGATGCACG
MD04G1164500 F	CTCTGAGGTCCGTTTCGACA
MD04G1164500 R	GATCTGCTCGTTTATGGCGG
MD10G1024000 F	GATGATGGGAGTCGGAGCTG
MD10G1024000 R	CCCACTGGAGCCAATCAACT
MD12G1178200 F	TATAGCCCCTCACCCCACTC
MD12G1178200 R	CCACTGCTTCGCTCGATACA
MD12G1178500 F	GTCGACGATGTCTCTGAGGG
MD12G1178500 R	TTAATGGCGGCCTCGGATTC
β-Actin F	GGATTTGCTGGTGATGATGCT
β-Actin R	AGTTGCTCACTATGCCGTGCT

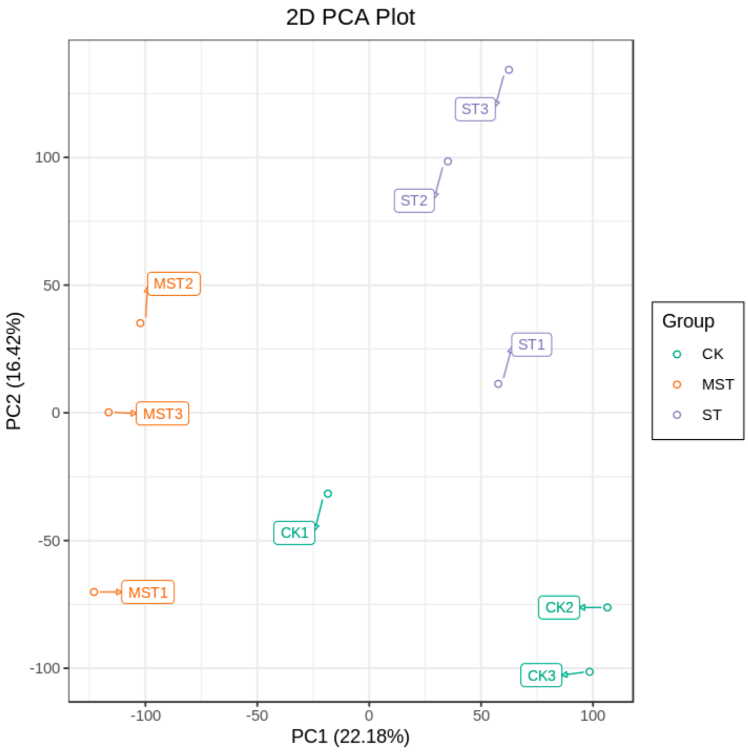

Figure. S1 Principal component analysis (PCA) of expressed genes.

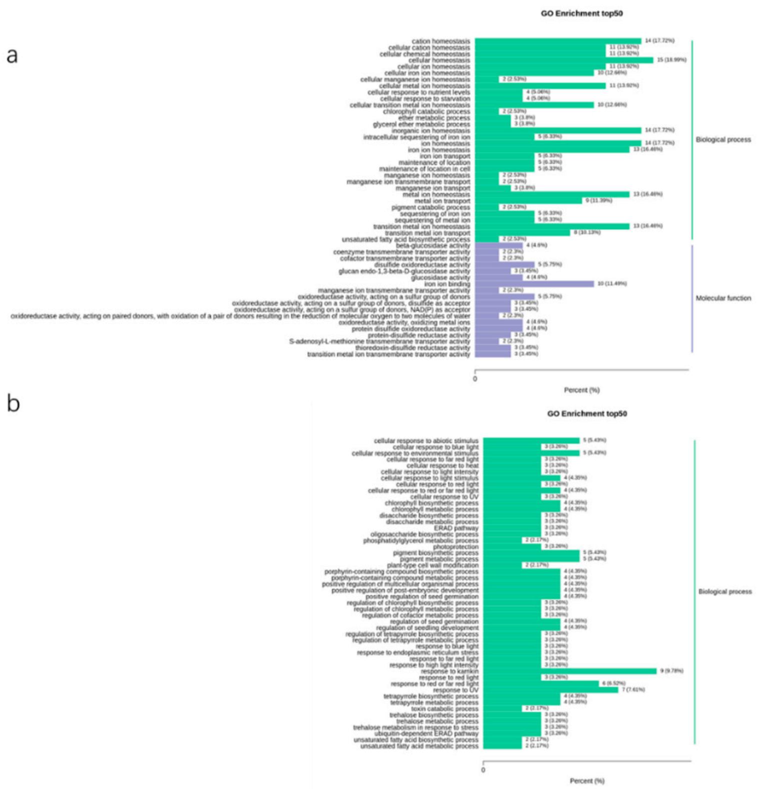

Figure. S2 GO annotation of DEGs in ST/CK (A) and MST/ST (B). The x-axis indicates the GO classifications, and the Y-axis indicates the number of genes in each classification.

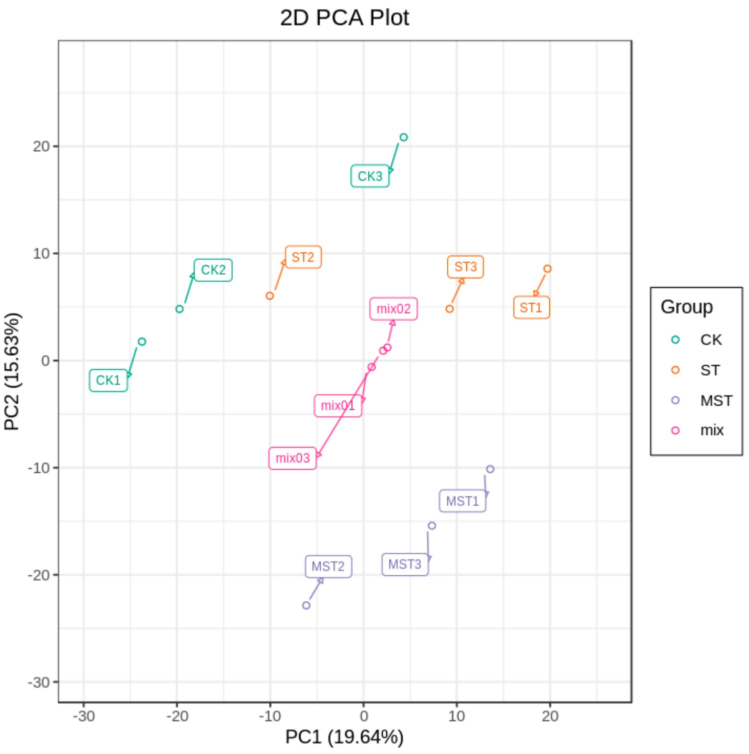

Figure. S3 Principal component analysis (PCA) of expressed metabolites.

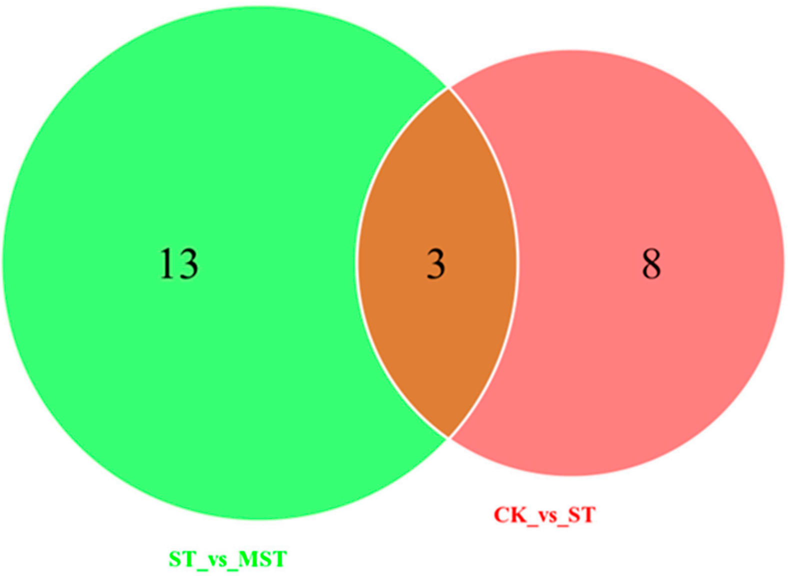

Figure. S4 Differential expressed metabolites in ST/CK and MST/ST.

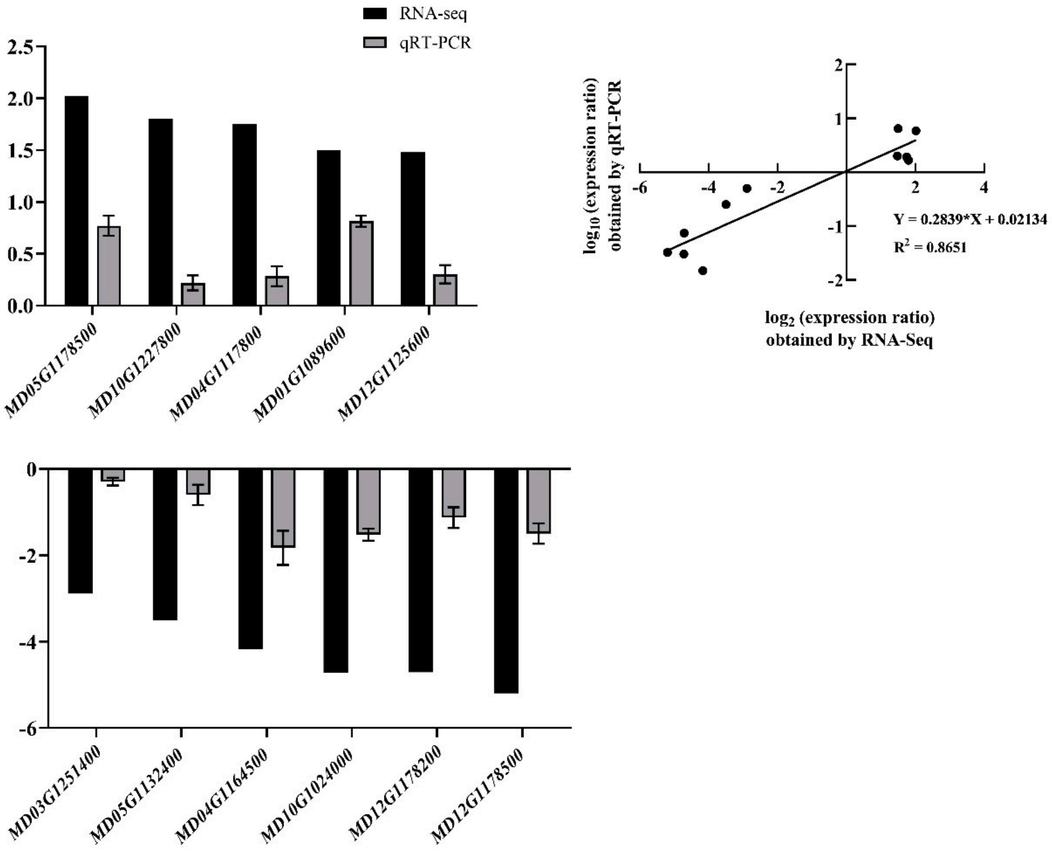

Figure. S5 qRT-PCR verification of DEGs, where black bars represent RNA-seq data (log_2FoldChange) and gray bars represent qRT-PCR data ($log_{10}2^{-\Delta\Delta CT}$).
